# Engineering the Future of ADCs in Non-Small Cell Lung Cancer

**DOI:** 10.32604/or.2026.078000

**Published:** 2026-06-16

**Authors:** Mi Rim Kim, Jason Lau, Michele Maffezzoli, Giuseppe Luigi Banna

**Affiliations:** 1 Oncology Unit, Portsmouth Hospitals University NHS Trust, Portsmouth, UK; 2 Department of Medicine and Surgery, University of Parma, Parma, Italy

**Keywords:** Antibody–drug conjugates (ADCs), non-small cell lung cancer (NSCLC), biomarker, linker, payload, bispecific

## Abstract

Antibody–drug conjugates (ADCs) are a promising strategy in non-small cell lung cancer (NSCLC), but early-generation drugs were limited by suboptimal target selection, heterogeneous drug–antibody ratios, and linker instability, resulting in modest efficacy and relevant toxicities. We performed a narrative review based on a literature search of PubMed and major oncology congresses up to October 2025, with the aim to critically analyzing the evolving biomarker landscape and engineering strategies shaping next-generation ADC development in NSCLC. Emerging approaches to identify targets for ADCs and refine patient selection include digital pathology with artificial intelligence technologies, transcriptomic and proteomic profiling, and liquid biopsy. Modern platforms incorporate site-specific conjugation technologies to achieve controlled and homogeneous drug-to-antibody ratio (DAR) distributions, improving pharmacokinetic predictability and reducing off-target effects. Advances in linker chemistry enhance plasma stability while preserving efficient intracellular payload release, balancing bystander effect with safety. These innovations are designed to enhance tumor selectivity, mitigate off-target toxicity and overcome resistance mechanisms. In conclusion, next-generation ADCs in NSCLC integrate refined biomarker strategies with advances in antibody engineering, linker design and payload biology. Emerging approaches, including immune-stimulating and bispecific ADCs, novel payloads, and combinations with tyrosine kinase inhibitors (TKIs) or immunotherapy, may improve efficacy, overcome resistance and expand the therapeutic window. Bispecific and dual-payload ADCs, as well as immune-stimulating ADCs, further aim to overcome resistance, exploiting both direct cytotoxicity and immune system activation and positioning ADCs as a crucial component of precision oncology in NSCLC.

## 1 Introduction

Antibody–drug conjugates (ADCs) are an emerging therapeutic class that works by delivering cytotoxic chemotherapy to cancer cells using a monoclonal antibody targeting a specific tumor antigen. In 2000, gemtuzumab-ozogamicin was approved, and so far, several ADCs have received regulatory approval for use in hematological and solid cancers [1]. However, early-generation ADCs were frequently limited by toxicities, insufficient efficacy and flawed biomarker selection, paving the way for a new generation of ADCs built on engineered antibodies and refined linkers [2]. Similarly, there has been emphasis on the importance of biomarker-driven patient selection to improve ADCs’ efficacy, targeting the core of tumor biology [3]. These insights led to the development of new ADCs that have marked great oncology milestones, like trastuzumab-deruxtecan (T-DXd) in breast cancer and enfortumab-vedotin in urothelial cancer [4,5,6]. 

However, in non-small cell lung cancer (NSCLC), ADCs struggled to find their way into clinical practice. Most trials in NSCLC tested ADCs against standard treatments in later lines in unselected populations, with no encouraging results [7]. In addition, despite initial encouraging outcomes, several early-phase trials were terminated due to unanticipated dose-limiting toxicities [8]. Recently, ADCs targeting Human Epidermal Growth Factor Receptor 2 (HER2), Trophoblast Cell Surface Antigen 2 (TROP2), and Mesenchymal-Epithelial Transition Factor (c-MET) demonstrated efficacy in biomarker-selected populations, further highlighting the need to design ADCs based on tumor biology, with greater attention to target selection [9]. It is therefore evident that future ADCs should rely on strategic engineering to optimize antigen selection, linker stability, payload potency, and interaction with the tumor microenvironment (TME), combining biologic, immunologic and targeted therapy [10]. 

In this scenario, the aim of this review is not to provide a list of existing evidence on ADCs in NSCLC, but rather to summarize the landscape of new biomarkers and describe the distinctive features of next-generation ADCs in NSCLC and their stage of clinical development. 

## 2 Search Strategy

This is a narrative review. A comprehensive literature search was conducted on PubMed and major oncology congress abstract repositories. The search was performed up to October 2025 using various combinations of the following keywords: “antibody–drug conjugates”, “ADC”, “non-small cell lung cancer”, “NSCLC”, “lung cancer”, “biomarkers”, “HER2”, “HER3”, “TROP2”, “bispecific ADC”, “next-generation ADC”, “engineering”, “payloads”, “emerging”, “linker”. In this review, we included original clinical trials, retrospective analyses, preclinical studies, systematic and narrative reviews relevant to ADCs in NSCLC. Articles were selected based on their relevance and level of evidence.

## 3 Biomarkers in ADCs: Limitations of Immunohistochemistry (IHC)

As we mentioned previously, biomarkers are crucial to identify patients most likely to respond, avoiding unnecessary toxicity. Regarding ADCs, biomarkers can also predict payload distribution, delivery and internalization [11]. Therefore, accurate characterization of antigen expression is fundamental. IHC is the most used method in routine pathology for assessing antigen expression on tumor cells and guiding eligibility for ADCs, such as for HER2. IHC is rapid and not expensive, but it also has some limitations. Firstly, antigen expression assessment is qualitative or semi-quantitative, and this poses several challenges. For instance, HER2 low and ultralow breast cancers can still respond to T-DXd, likely due to the bystander effect, which is broadening eligibility to low expressor populations. This means that an all-or-none (positive vs. negative) cutoff is no longer sufficient. Low levels of antigen expression require defining actionable thresholds, while high expression requires distinguishing true oncogenic drivers from highly expressed but biologically irrelevant antigens. Intermediate expression further complicates clinical decision-making, often supporting combination strategies or further testing [11,12]. Secondly, there is substantial analytical and interpretive variability: differences in antigen retrieval, antibody clone, instrumentation, and pathologist interpretation can mean that IHC results are not always reproducible [13]. Thirdly, intra- and inter-lesional heterogeneity impacts ADC target expression, leading to uneven binding and reduced efficacy of ADCs [14]. Antigen expression is dynamic and can change over time with treatment or across metastatic sites; hence, IHC may not reflect the current target expression at the time of ADC administration [14]. Lastly, the role of antigen expression with ADCs is not universally predictive. Indeed, approvals for different ADCs came regardless of IHC expression (e.g., TROP2, Nectin-4) [12]. Altogether, these limitations suggest that relying on IHC alone may lead to misclassification of patients, even though IHC still remains the backbone of biomarker testing. These limitations highlight the need for new approaches to identify biomarkers for selecting patients. 

## 4 Emerging Biomarker Identification Approaches

### 4.1 Digital Pathology

Emerging biomarker identification strategies are now designed to overcome limitations of IHC (Table 1). An ideal target should exhibit high tumor-specific expression with limited normal tissue expression, allowing for a homogeneous distribution within the tumor and efficient internalization after binding. In addition, it should reflect relevant tumor biology, be reproducible and easy to measure, capture temporal dynamics of disease evolution and demonstrate a strong predictive value for therapeutic response [15]. Unfortunately, such ideal targets do not exist in clinical practice, although novel quantitative assessments are in development using transcriptomic, liquid biopsy, immunofluorescence-based assay, mass spectrometry, and digital pathology [16]. These assays can also be integrated with machine learning and artificial intelligence (AI) technologies, which have the potential to capture intra-tumor antigen gradients, overcome interobserver variability and reduce time expenditure, having shown a strong concordance with expert pathologists [17]. AI can also infer multiplex phenotypes from routine hematoxylin and eosin (H&E) by learning from multi-omics and imaging data, thus predicting biomarker status [18,19]. Similarly, digital pathology can refine patient stratification by capturing spatial variability of antigen expression and determining whether the expression is membranous or cytoplasmic, as well as the degree of heterogeneity in the sample [20,21]. Indeed, digitalized TROP2 IHC-stained whole-slide images (WSI) of tissue samples from patients with NSCLC have been used to develop the Quantitative Continuous Scoring (QCS) model, a deep learning algorithm trained on pathologists’ annotations to identify tumor areas and cellular compartments (membrane and cytoplasm) of each tumor cell across the WSI [22]. The QCS model measured TROP2 expression in the membrane relative to the cytoplasm, resulting in a normalized membrane ratio (NMR). In this sub-analysis of the TROPION-Lung01, datopotamab-deruxtexan (dato-DXd) demonstrated greater efficacy in patients with TROP2 QCS-NMR positive non-squamous (n-sq) metastatic NSCLC without actionable genomic alterations (AGA), demonstrating potential as a predictive biomarker. Notably, TROP2 QCS-NMR positivity was higher in patients with n-sq histology [22]. 

### 4.2 Radioligands

An additional strategy could involve molecular functional imaging with radiolabeled antibodies, which can enable the visualization of biomarker expression throughout the body. This strategy can assess TROP2 expression in real-time and non-invasively, offering guidance for targeted therapies. Studies have shown that TROP2-targeted radiotracers exhibited superior imaging performance in TROP2-positive tumors compared to stand-of-care examinations, demonstrating great clinical potential [23]. 

### 4.3 Transcriptomic and Proteomic Profiling

Another key approach is transcriptomic and proteomic profiling of tumor biology, including drug efflux and DNA-repair pathway activation, that can shape ADC susceptibility but may be invisible to IHC. Integrating these layers into models of disease progression and therapy response may discover biomarkers that go beyond just the presence or absence of an antigen [24]. Transcriptomic profiling can capture resistance pathways better than IHC. For instance, researchers analyzed the transcriptomic tumor profile of six drug efflux pump genes generated from pre-treatment biopsies collected from patients with metastatic breast cancer (MBC) before ADC treatment to elucidate biomarkers of response and resistance to sacituzumab-govitecan (SG) and T-DXd [25].

### 4.4 Circulating Tumor DNA

A further strategy is represented by circulating tumor DNA (ctDNA) and circulating tumor cells (CTCs) assessed through liquid biopsy, which can track dynamic tumor evolution (antigen loss, clonal selection, and resistance mechanisms) across tumor sites at different time points [12,24]. Patients responding to SG have significantly higher TROP2–positive CTCs at baseline than non-responding patients. Also, the number of TROP2–positive CTCs in responder patients significantly decreased during treatment. Separately, ctDNA analysis found a patient with a *TOP1* gene mutation acquired during progression on SG, confirming that *TOP1* gene mutations can lead to resistance to SG, as demonstrated in MBC [26]. 

**Figure 1:**
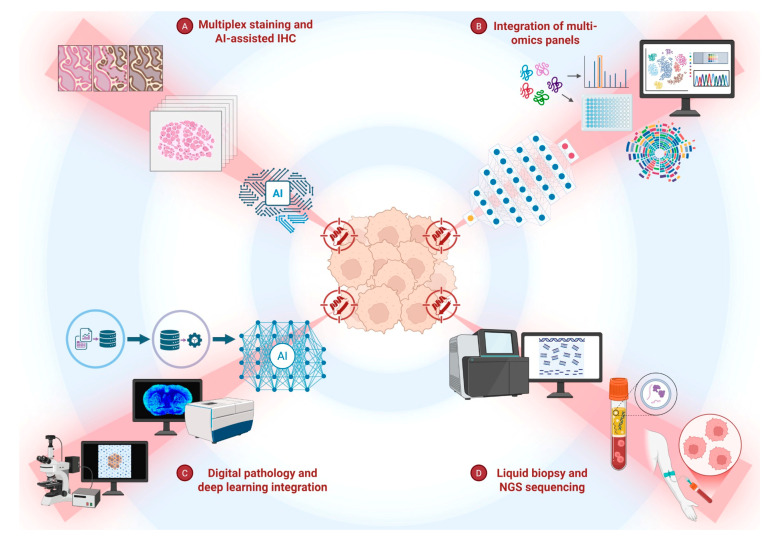
Multimodal biomarker strategies to support biomarker discovery and patient selection for ADC development. (**A**) Multiplex staining and AI-assisted IHC. (**B**) Integration of multi-omics platforms (transcriptomics, proteomics, genomics) with machine learning model. (**C**) Digital pathology with deep learning–based quantification. (**D**) Liquid biopsy and NGS-based ctDNA and CTCs profiling for longitudinal monitoring. This figure was created with Biorender. Abbreviations: ADC = antibody-drug conjugate; AI = artificial intelligence; CTC = circulating tumor cell; ctDNA = circulating tumor DNA; IHC = immunohistochemistry; NGS = next-generation sequencing.

In summary, future development of biomarkers for ADCs will likely require multi-modal approaches integrating IHC, transcriptomics, and liquid biopsy with the help of AI-based technologies for patient selection and monitoring of treatment response and resistance. However, challenges remain, including accessibility to biomarker testing and disparities in diagnostic infrastructure [18]. Therefore, equally important will be the collaboration between oncologists, pathologists, and bioinformaticians to accelerate translation into clinical practice and personalize ADC therapy [18]. A summary of the emerging biomarker workflow is reported in Fig. 1.

**Table 1:** Evolution of biomarker strategies in ADC development.

Method	Strengths	Limitations	Emerging Enhancement	Clinical Application	Emerging Enhancements
Immunohisto-chemistry (IHC)	Widely available, morphological details, low cost	Semi-quantitative scoring misses low expressors and tumor heterogeneity/tumor dynamics, observer/lab variability	AI-assisted scoring, integration with multiplex staining	Routine HER2 amplification assessment	Target selection based on antigen expression
Digital Pathology + AI Quantification	Standardized scoring, membranous/cytoplasmic gradients detection, reproducible	Requires algorithm training and large datasets, with potential bias if trained on limited populations	Deep learning integration with H&E and IHC for phenotype prediction	TROP2 QCS-NMR expression [22]	Detection of tumor antigen spatial heterogeneity to support use of ADCs
Transcriptomic and Proteomic Profiling	Quantitative assessment captures function and tumor biology	Complex analysis, expensive; not yet standardized for clinical decision-making	Integration with multi-omics panels	Transcriptomic profiling of drug efflux pumps [25]	Enables ADC selection based on tumor functional biology not only on antigen expression
Liquid Biopsy (ctDNA/CTCs)	Non-invasive, real-time monitoring of tumor evolution	Sensitivity challenges, low tumor DNA yield, standardization issues	Multi-omics ctDNA and AI tracking for adaptive therapy	ctDNA detection of TOP1-mediated ADC resistance [26]	Detects antigen loss and acquired resistance to dynamically guide ADC selection

Abbreviations: ADC = antibody-drug conjugate; AI = artificial intelligence; CTC = circulating tumor cell; ctDNA = circulating tumor DNA; H&E = hematoxylin and eosin; HER2 = human epidermal growth factor receptor 2; TROP2 = trophoblast cell surface antigen 2; QCS = quantitative continuous scoring; NMR = normalized membrane ratio.

## 5 Refining ADC Biology: From Structural Optimization to Functional Precision

### 5.1 Drug-to-Antibody Ratio (DAR) and Conjugation Strategies

An ideal ADC should combine a highly specific antibody for a tumor-specific target with efficient internalization; the linker should be stable in the bloodstream, ensuring controlled intratumoral payload release; eventually, the payload should be sufficiently potent, chemically optimized to avoid aggregation, with high conjugate homogeneity and appropriate DAR to prevent off-target clearance and toxicity [27]. An appropriate DAR is particularly important because ADCs with a low DAR may not achieve the desired clinical efficacy, while ADCs with a high DAR may achieve higher plasma concentrations and exhibit greater off-target toxicity [28]. Consequently, variability in DAR has implications in terms of predictable pharmacokinetic profiles, efficacy and tolerability in real world settings. Beyond the numerical value of DAR, the distribution of DAR within an ADC critically determines its behavior. Early ADCs were made by non-site specific conjugation, which was accomplished by modifying lysine **ε**-amines or interchain cystine thiols in the conjugation site. This produced mixtures of molecules with variable DAR values ranging from 0 to 8 and a broad polydispersity index (PDI). A higher PDI reflects a wider particle size and molecular distribution, which is associated with reduced stability, increased aggregation and variable clearance [29,30]. This structural variability modifies solubility, charge distribution and hydrophilic–lipophilic balance, thereby affecting ADC performance: high DARs tend to exhibit increased hydrophobicity and aggregation, faster plasma clearance and hepatic uptake, whereas low DARs were less potent. Furthermore, structural variability can promote the formation of ADC immune complexes, thus accelerating clearance and further compromising pharmacokinetic stability [31]. The resulting early ADCs displayed unpredictable pharmacokinetics and narrow therapeutic windows that threatened clinical utility. For example, gemtuzumab-ozogamicin, an early ADC used to treat acute myeloid leukemia, was withdrawn from the market due to safety concerns attributed to its broad DAR distribution, unstable hydrazone linker, and highly potent payload calicheamicin, demonstrating the importance of reducing DAR variability [31].

DAR distribution is deeply connected to the conjugation strategy. Early lysine coupling was associated with greatly variable DARs because each IgG1 antibody typically possesses more than 20 potential attachment sites. In contrast, cysteine coupling was preferred because IgG1 and IgG4 antibodies possess four pairs of interchain disulfide bonds, enabling greater precision in conjugation [15]. However, conjugation remains partially stochastic when native interchain disulfides are reduced, still generating a broad distribution of DAR species. Taking into account considerations such as chemo- and regio-selectivity, additional methods were sought to enable controlled attachment of measured payload to specific positions on antibodies to create homogenous ADCs with consistent DARs, which brought about site-specific conjugation methods [30]. Such methods include engineered cysteine residues that provide selective conjugation sites, unnatural amino acid incorporations for precise coupling, and enzymatic ligation methods to create site specific attachment points. Engineered cysteine approaches introduce defined reactive thiol residues, although introduction of free sulfhydryl groups may predispose to unintended disulfide scrambling, requiring careful structural validation. Unnatural amino acid coupling introduces non-natural amino acids into the antibody sequence, allowing precise drug attachment and homogenous DAR. However, the potential immunogenicity of non-natural residues can carry a greater risk of immune response [31]. Enzymatic ligation strategies employing transglutaminase, formylglycine-generating enzyme or Sortase A enable selective modification of engineered sequences on the antibody structure. These approaches achieve regio-selective conjugation with minimal off-target modification, resulting in homogeneous ADCs with narrow DAR distributions and reduced physicochemical variability [31]. In parallel, click chemistry reactions (e.g., strain-promoted azide–alkyne cycloaddition) allow rapid, high-yield and stereoselective coupling under mild conditions, minimizing structural variation of the antibody and contributing to improved stability [31]. Collectively, these site-specific methods shift ADC engineering from a stochastic conjugation model toward a controlled molecular architecture, resulting in the development of more stable ADCs with improved efficacy [30]. 

### 5.2 Advancements in Target Selection and Linker Design

Historically, ADCs were designed to target tumor antigens with high expression in tumor cells (e.g., HER2), but both antigen heterogeneity and target affinity may influence efficacy and safety [32]. High antibody affinity may improve tumor uptake but also increase the likelihood of normal tissue trapping in cases of shared antigen expression, thus increasing toxicity. The field has therefore moved toward prioritizing truly tumor-restricted antigens and refining affinity thresholds to balance penetration and selectivity [32]. 

In parallel, linker design has also evolved. Indeed, ADC toxicity seemed to be more related to linker stability and payload rather than to the antibody specificity [33]. Fu et al. and Wang et al. further supported that the chemical property of payloads (hydrophobicity, membrane permeability, and bystander effect) can affect efficacy, with hydrophobic payload and linker combinations leading to aggregation, clearance and narrow therapeutic windows, thus emphasizing the importance of payload and linker selection to achieve a better therapeutic index [1,31]. In contrast, hydrophilic linkers can improve stability, pharmacokinetics and reproducibility, thus reducing inter-patient variability and expanding the therapeutic window [31]. Linker advancements have also evolved from cleavage prone linkers that led to early plasma release to a modern linkers with better plasma stability and triggered payload release only in the tumor or after antibody internalization [34]. Bargh et al. classified cleavable linkers based on their cleavage mechanisms: acid cleavable, which exploits endosomal or lysosomal pH-like hydrazones, reduction-sensitive disulfides that respond to intracellular glutathione concentration, enzyme cleavable dipeptides relying on lysosomal proteases to enable selective intracellular release. Cleavable linkers enable payload release and potent bystander effect but may lead to off-target release and systemic toxicity [34]. On the other hand, non-cleavable linkers keep the drug attached until antibody degradation, giving better plasma stability but requiring full internalization and degradation, thus limiting efficacy in antigen-low tissues [34]. These considerations underscore why next-generation ADCs should improve linker selection by integrating tumor biology [33].

### 5.3 Payload Properties and Determinants of Toxicity

The payload choice remains central in ADC performance. Early payloads used tubulin-disrupting agents like maytansinoids (DM1) and monomethylauristatin (MMAE), which had potent cytotoxicity but were limited by systemic toxicity and resistance mechanisms such as upregulation of efflux pumps [35]. The introduction of topoisomerase I (topo I) inhibitor payloads, such as DXd, has dramatically revolutionized ADCs. Metabolites from these payloads are membrane permeable with a more potent bystander effect that can kill neighboring tumor cells with low or absent antigen levels, thus addressing tumor heterogeneity [36]. The spheroid imaging data, a sensitive platform using tumor spheroids and a pharmacodynamic marker to quantitatively map the penetration of cytotoxic bystander payloads, showed that payloads like MMAE and calicheamicin D had a deeper penetration within the spheroid compared to DM1 and calicheamicin G which only affected the targeted cells and those immediately adjacent to it [37]. Khera et al. also described how to quantify the theoretical bystander penetration of payloads by capturing the ratio of cell uptake versus tissue diffusion of payload, to predict optimal payload physicochemical properties [37]. Payload penetration within cells also depends on lipophilicity, charge, hydrogen bonding capacity and molecular weight. Greater permeability can increase uptake by bystander cells, but if too high can fail to reach deeper regions, and if low, the payload would be washed out before achieving intracellular cytotoxic concentrations. Hence, new ADCs should address both the chemical properties of membrane permeability and the optimal radius of payload effect needed for effective bystander killing of tumor cells [37].

**Figure 2:**
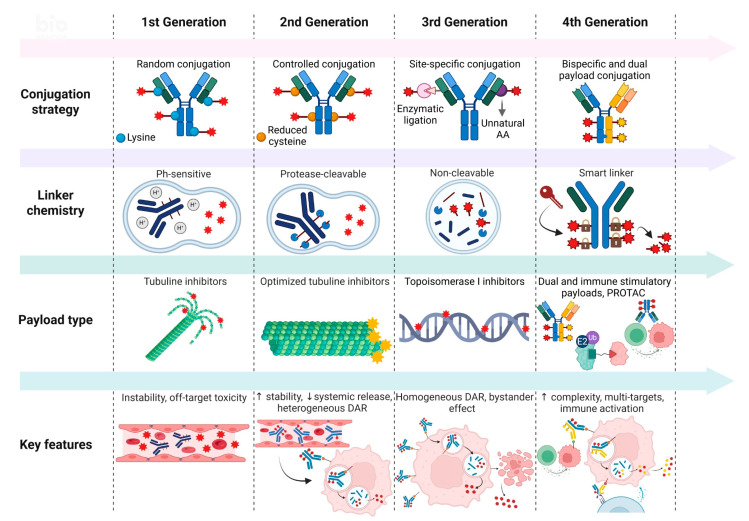
Comparative representation of the four generations of ADCs, highlighting advances in conjugation strategy, linker chemistry, payload type and key biological features. First-generation ADCs relied on random conjugation and acid-labile linkers, resulting in blood instability and off-target toxicity. Second-generation ADCs introduced controlled cysteine conjugation and protease-cleavable linkers, improving stability but with heterogeneous DAR. Third-generation ADCs employ site-specific conjugation, non-cleavable linkers binding topoisomerase I inhibitor payloads, enabling homogeneous DAR and bystander killing. Fourth-generation ADCs integrate bispecific antibodies, dual payloads, smart linkers, and immune-stimulatory or PROTAC payloads, increasing mechanistic complexity. This figure was created with Biorender. Abbreviations: ADC = antibody-drug conjugate; DAR = drug-antibody ratio; PROTAC = PROteolysis TArgeting Chimera.

However, the future of payloads extends beyond traditional cytotoxic agents and is heading towards immune-modulating and PROteolysis TArgeting Chimera (PROTAC) payloads, transforming ADCs into multimodal agents for direct tumor killing and immune system activation [38,39]. A summary of the modifications across ADC generations is reported in Table 2 and Fig. 2. 

Eventually, off-target toxicities remain a major limitation. ADC-related toxicities can arise from target-dependent and target-independent mechanisms, and are strongly influenced by the properties of the payload, linker and antibody. While low level expression of the target in normal tissues can result in receptor-mediated uptake and toxicity, this is often poorly predictive of ADC safety. Instead, target-independent mechanisms, including non-specific endocytosis, Fc receptor–mediated uptake, premature linker cleavage, bystander effect and systemic release of payloads, play a crucial role in many toxicities [40]. For example, neutropenia is a common toxicity of ADCs largely caused by premature payload release due to linker instability and subsequent bystander effect on hematopoietic cells. The cytotoxic effect of MMAE was noted in differentiating neutrophils, likely mediated by serine proteases mediating extracellular cleavage, while, in patients with colorectal cancer, decreased SN-38 glucuronidation was associated with neutropenia. Ocular toxicity is thought to be driven by macropinocytosis in corneal epithelial cells, independent of antigen expression. Similarly, hepatic toxicity and thrombocytopenia have been associated with non-specific clearance mechanisms involving Kupffer cells, sinusoidal endothelial cells, Fc receptor–expressing cells, and possibly mannose receptor–mediated uptake [40]. FcγR-mediated uptake into differentiating megakaryocytes has been implicated in thrombocytopenia with T-DM1, despite a lack of HER2 expression, highlighting the contribution of Fc-dependent internalization to hematologic off-target toxicity. T-DXd, associated with interstitial lung disease (ILD), was primarily localized in alveolar macrophages but not pulmonary epithelial cells, again suggesting target-independent uptake [40].

In order to minimize adverse effects, it is especially important to ensure predictable pharmacokinetics and payload-linker stability. Linker instability, high DARs, and increased hydrophobicity enhance systemic payload exposure and non-specific clearance, reinforcing the direct link between ADC engineering and toxicity patterns. Comparisons of ADCs with cleavable and non-cleavable linkers have shown reduced toxicity with non-cleavable linkers for specific targets [31]. While increased membrane permeability can augment the bystander effect in ADCs and improve tumor killing, the increased cellular permeability required to achieve this effect can worsen off-target toxicity. Many drug-linker combinations are hydrophobic, and in agents with a high DAR, this can contribute to ADC aggregation and non-specific clearance by Kupffer cells [31]. Advancements in ADC engineering aimed at reducing these toxicity mechanisms while preserving antitumor efficacy. As stated previously, the development of more stable linkers to minimize premature payload release, optimization of DAR to reduce hydrophobic clearance, selection of payload with reduced membrane permeability to limit bystander toxicity, as well as site-specific conjugation technologies, can effectively reduce off-target toxicities [15]. In parallel, conditionally active ADCs, particularly probody therapies utilizing masking peptides that are exclusively cleaved by tumor-specific proteases, are able to restrict payload release to the TME [15]. Additional engineering solutions include Fc modifications to reduce hepatic uptake and modulation of surface charge to limit non-specific endocytosis, further highlighting how ADC design is evolving toward complex integrated platforms [40]. 

**Table 2:** Timeline of ADCs development across generations.

ADC Generation	Conjugation Strategy	Linker Chemistry	Payload Type	Key Improvement	Limitations
1st Generation	Random conjugation	Acid-labile hydrazone; reducible disulfide linkers	Tubulin inhibitors (DM1, MMAE)	Not applicable	Poor stability, variable DAR, off-target toxicity, narrow therapeutic window [35]
2nd Generation	Controlled conjugation (partial cysteine reduction)	Protease-cleavable	Refined tubulin inhibitors	Improved linker stability, internalization efficiency, and reduced systemic release	Heterogeneous DAR, limited payload diversity [36]
3rd Generation	Site-specific conjugation	Highly stable cleavable and non-cleavable linkers	Topoisomerase I inhibitors, DNA-damaging payloads	Homogeneous DAR, bystander effect, improved pharmacokinetics, controlled payload release	Residual resistance due to antigen loss or efflux, need for biomarker integration [36]
4th Generation (Emerging)	Bispecific and dual-payload conjugation	Stimulus-responsive linkers (pH-, enzyme-, or redox-sensitive)	Dual cytotoxic payloads, immune stimulatory payloads, PROTACs	Multi-target binding, immune activation, overcoming resistance, and broadening indications	Complexity of manufacturing, biomarker dependence [38,39]

Abbreviations: ADC = antibody-drug conjugate; DAR = drug-antibody ratio; MMAE = monomethyl auristatin E; PROTAC = PROteolysis TArgeting Chimera; DM1 = derivative of maytansine 1.

## 6 A Brief Landscape of Current ADCs in NSCLC

The development of ADCs in NSCLC has rapidly expanded over the past decade. Currently, three ADCs have received Food and Drug Administration (FDA) approval in patients with previously treated advanced NSCLC: T-DXd for patients with HER2-mutant NSCLC, telisotuzumab vedotin (teliso-V) for c-MET–overexpressing, Epidermal Growth Factor Receptor (EGFR)-wild-type NSCLC, and dato-DXd for EGFR-mutated NSCLC after prior EGFR-directed therapy and platinum-based chemotherapy. Several targets have been explored, leading to the development of different ADCs, which currently remain under clinical investigation, Table 3 [9]. 

TROP2 is a cell surface glycoprotein that has been associated with cancer cell growth and proliferation. It is expressed in many epithelial cancer cells and has been a target of interest for ADCs. Two different ADCs targeting TROP2, SG, and dato-DXd have been developed. SG consists of sacituzumab, an anti-TROP2 monoclonal antibody, linked to SN-38, a topoisomerase I inhibitor, via a hydrolyzable cleavable linker. Dato-DXd consists of datopotamab, another anti-TROP2 antibody, linked to DXd, a topoisomerase I inhibitor, by a peptide cleavable linker. Both SG and dato-DXd have been tested in phase III trials in patients with NSCLC progressing on/after platinum-based chemotherapy, anti–Programmed Cell Death Protein (Ligand) 1 (PD-[L]1), and targeted treatment for AGA tumors, in comparison with the standard chemotherapy, docetaxel. Both drugs demonstrated no statistically significant improvement in overall survival (OS) [41,42]. Notably, recent data confirmed that TROP2 expression by IHC does not predict response, underscoring the complexity of biomarker development for ADCs, as previously mentioned [22]. In the TROPION-Lung01 study, efficacy appeared enhanced in adenocarcinoma relative to squamous histology, suggesting potential histotype-specific sensitivity [42]. Interestingly, the phase Ib TROPION-Lung02 trial is evaluating dato-DXd combined with pembrolizumab with or without platinum agents in both previously untreated and pretreated patients with metastatic NSCLC without AGA. In 33 evaluable patients, the objective response rate (ORR) was 54% (62% with doublet and 50% with triplet therapy) [43]. 

Patritumab-deruxtecan is an ADC targeting HER3, whose overexpression is a key resistance pathway in EGFR-mutated NSCLC. It consists of an anti-HER3 monoclonal antibody linked to DXd by a cleavable tetrapeptide linker. Although HER3 may have weaker kinase activity and limited signaling capacity, and therefore may be a less effective target compared to EGFR or HER2, the conjugation of an anti-HER3 antibody to a topoisomerase I inhibitor could facilitate cytotoxin delivery and tumor cell death [44]. Unfortunately, trials in patients after EGFR tyrosine kinase inhibitor (TKI) therapy led to the same somewhat disappointing results. After promising results in early phase trials, where it achieved a response rate of 30% and a median progression-free survival (mPFS) of 5.5 months in patients pretreated with EGFR-TKI and chemotherapy, the recent HERTHENA-Lung02 study showed a lack of OS benefit compared to chemotherapy in the post-EGFR-TKI setting [45,46]. 

The breakthrough came with T-DXd, an anti-HER2 antibody conjugated to DXd by a protease-cleavable peptide linker. T-DXd is characterized by high membrane permeability and has a cleavable linker, which allows for greater bystander effect [47]. The phase II DESTINY-Lung01 study evaluated T-DXd in NSCLC harboring HER2 overexpression and HER2 mutations. In the updated analyses reported by Li et al., T-DXd showed an ORR of 55%, a median PFS and mOS of 8.2 and 17.8 months, respectively [48]. In the DESTINY-Lung02 trial, T-DXd was administered to patients who had previously been treated for metastatic HER2-mutant NSCLC with an associated ORR of 49–56%. The results of the DESTINY-Lung01 and Lung02 trials led to the FDA approval for patients with previously treated HER2-mutant NSCLC [49]. DESTINY-Lung04 (NCT05048797) is currently investigating the efficacy of T-DXd as a first-line treatment in patients with metastatic NSCLC harboring HER2 mutations. Interestingly, the DS8201-A-U106 study of T-DXd and pembrolizumab in treatment-naive patients with HER2-expressing or HER2-mutated NSCLC yielded an ORR of 55% to 67%, with interstitial lung disease occurring in 11 (20%) of 55 patients [50]. 

These heterogeneous outcomes likely reflect differences in target biology, biomarker selection and prior treatment exposure, limiting optimal patient selection. The favorable results achieved with T-DXd likely reflect the biological role of HER2 mutation, and to a lesser extent amplification, in NSCLC, together with the high DAR and potent bystander effect of T-DXd. Nonetheless, more sensitive and discriminative diagnostic platforms are needed [9]. In contrast, TROP2-directed ADCs, as well as CEACAM5-targeted agents, yielded modest or negative results. For TROP2 ADCs, this can be related to several factors: IHC is not predictive of response, whereas more sophisticated metrics, such as the NMR, appear more informative. Notably, Dato-DXd showed consistent benefit in n-sq NSCLC, particularly in EGFR-mutant tumors: this finding may be explained by higher and more homogeneous TROP2 expression in EGFR-mutant tumors, enhanced EGFR-driven clathrin-mediated endocytosis and the robust bystander effect caused by DXd within dense EGFR-mutant tumor clusters [51]. In contrast, SG and novel TROP2-directed ADCs may retain activity in squamous tumors, particularly in PD-L1–enriched contexts [9]. Similar considerations can be extended to other negative trials: the absence of a robust predictive biomarker beyond EGFR mutation status for patritumab-deruxtecan and the lack of predictive significance of CEACAM5 for tusamitamab ravtansine despite the selection of patients with high expression of CEACAM5 [45,52,53]. Collectively, these data indicate that ADC efficacy in NSCLC is dependent on appropriate patient selection, which will likely require advanced biomarker selection strategies, as mentioned previously. Meanwhile, the clinical impact of ADCs will likely be maximized through combinations with immune checkpoint inhibitors (ICIs) or other agents [9]. In line with this concept, multiple combination strategies are actively being explored and will likely change clinical practice in selected subpopulations. Dato-DXd is being evaluated with ICIs and/or chemotherapy in multiple trials (TROPION-Lung02/07/08) [43,54,55]. T-DXd is under investigation in the first-line setting in patients with HER2-expressing or HER2-mutant NSCLC, including DESTINY-Lung04 (NCT05048797) and a phase Ib study with pembrolizumab [50]. In parallel, phase III trials of novel TROP2-directed ADCs (SKB264 and MK2870) combined with pembrolizumab (NCT06448312, NCT06170788) are directly challenging current standards in PD-L1–high NSCLC.

**Table 3:** ADCs in NSCLC.

Target	Drug Name	Phase	Patient Population	Previous Cancer Therapies	Key Efficacy Results	Key Safety/Status Notes
**HER2**	Trastuzumab Deruxtecan (T-DXd)	II (DESTINY-Lung01) [48]	Pretreated metastatic HER2-mutant NSCLC	≥2 L, median (range): 2 (1–7)	ORR: 55%; PFS: 8.2 mo; OS: 17.8 mo	Terminated; Grade ≥3 AEs: 46%; ILD: 26%
		II (DESTINY-Lung02) [49]	Pretreated metastatic HER2-mutant NSCLC	≥2 L, median (range): 2 (1–12)	ORR: 49%	Completed
		III (DESTINY-Lung04)	HER2-mutant NSCLC	None (1 L)	-	Recruiting
	T-DXd + Pembrolizumab	Ib (DS8201-A-U106) [50]	HER2-expressing or HER2-mutant NSCLC	None (1 L)	ORR: 55% (exp), 67% (mut); PFS: 15.1 mo (exp), 11.3 mo (mut)	Active, not recruiting; ILD in 11 pts (20%)
**HER3 (ERBB3)**	Patritumab Deruxtecan (HER3-DXd)	II (HERTHENA-Lung01) [46]	EGFRm NSCLC post-EGFR TKI & chemo	≥2 L, median (range): 3 (1–11)	ORR: 30%; PFS: 5.5 mo; OS: 11.9 mo	Completed
		III (HERTHENA-Lung02) [45]	EGFRm NSCLC post-EGFR TKI (vs. chemo)	2 L after third generation EGFR TKI	PFS: 5.8 mo; OS: 16 mo vs. 15.9 mo (NS)	No significant OS benefit
**TROP2**	Sacituzumab Govitecan (SG)	III (EVOKE-01) [41]	Metastatic NSCLC (vs. Docetaxel)	≥2 L, after PBC ± ICI; ≥1 prior TKI if AGA	OS: 11.1 vs. 9.8 mo (NS)	Active, not recruiting; No significant OS benefit
	Datopotamab Deruxtecan (Dato-DXd)	III (TROPION-Lung01) [42]	Metastatic NSCLC (vs. Docetaxel)	≥2 L after PBC + ICI; 1–2 prior TKIs + PBC ± ICI if AGA	PFS: 4.4 vs. 3.7 mo; OS: 12.9 vs. 11.8 mo (NS)	Active, not recruiting; Improved PFS, no significant OS benefit; better in adenocarcinoma
**c-MET**	Telisotuzumab Vedotin (Teliso-V)	II (LUMINOSITY) [56]	EGFR-wt, c-MET overexpressing NSCLC	≥2 L, median (range): 1 (1–3)	ORR: 28.6% (34.6% in IHC-high); PFS: 5.7 mo	Active, not recruiting; FDA approved May 2025
		I/Ib [57]	EGFRm, c-MET + NSCLC (Teliso-V + Erlotinib)	≥2 L after EGFR TKI	ORR: 32.1%; PFS: 5.9 mo	
		I/Ib [58]	EGFRm, c-MET+ NSCLC (Teliso-V + Osimertinib)	≥2 L after Osimertinib	ORR: 50%; PFS: 7.4 mo	
**PTK7**	Cofetuzumab Pelidotin (Cofe-P)	Ib [59]	PTK7-expressing recurrent NSCLC	≥2 L, median (range): 2 (1–4)	ORR: 18%; PFS: 5.5 mo; OS: 12.6 mo	Active, not recruiting
**B7-H3**	Ifinatamab Deruxtecan (I-DXd)	I/II [60]	Squamous NSCLC	≥2 L, previously treated	ORR: 31%	Ongoing
		II (IDeate-Lung01) [61]	Extensive-stage SCLC	≥2 L, median (range): 2 (1–3)	ORR: 48%; PFS: 4.9 mo	Active, not recruiting; ILD: 12%
**Integrin-β6**	Sigvotatug Vedotin (SV) + Pembro	I [62]	NSCLC (PD-L1 TPS ≥1%)	≥2 L, previously treated	ORR: 57% (n = 7)	Ongoing; Pneumonitis/ILD: 9.7%
**Transferrin Receptor-1 (TfR1/CD71)**	CX-2029	I/II [63]	Solid tumors (incl. NSCLC)	≥2 L, median (range): 3 (1–16)	PR: 50% (n = 4)	Completed; Protease-activated ADC
**AXL**	Enapotamab Vedotin (EnaV)	IIa [64]	Advanced treatment-refractory NSCLC	Previously treated not candidates for standard therapy	ORR: 19%	Development discontinued
	Mecbotamab Vedotin (BA3011) ± Nivolumab	II [65]	Metastatic non-squamous NSCLC	≥2 L, median (range): 3 (1 to ≥4)	ORR: 28%; DCR: 56% (alone)	Completed; CAB-AXL-ADC
**FOLR1 (FRα)**	Farletuzumab Eribulin (MORAb-202)	I [66]	FRα-positive solid tumors	≥2 L, median (range): 3 (1 to ≥3)	ORR: 41%	ILD: 23%
	Rinatabart Sesutecan (Rina-S)	I/II [67]	Selected solid tumors (incl. NSCLC)	Previously treated	-	Recruiting
**CEACAM5**	Tusamitamab Ravtansine	III (CARMEN-LC03) [53]	Non-squamous NSCLC, CEACAM5-high (vs. Docetaxel)	≥2 L, range 1–2, after PBC ± ICI; ≥1 prior TKI if AGA	OS: 12.8 vs. 11.5 mo (NS)	Active, not recruiting
		II (CARMEN-LC04) [52]	Non-squamous NSCLC, CEACAM5-high (Tus-Rav + Ramucirumab)	≥2 L, after PBC ± ICI; ≥1 prior TKI if AGA	ORR: 19%; PFS: 5.7 mo	Terminated
**NaPi2b**	Lifastuzumab Vedotin (LIFA)	I [68]	NSCLC	Previously treated not candidates for standard therapy	ORR: 8% (at active doses)	Completed
	Upifitamab Rilsodotin (UpRi)	I [69]	NSCLC adenocarcinoma & ovarian cancer	Previously treated not candidates for standard therapy	ORR: 23% (34% in NaPi2b-high)	Active, not recruiting
**Tissue Factor (CD142)**	Tisotumab Vedotin	I/II (InnovaTV 201) [70]	Selected solid tumors (incl. NSCLC)	≥3 L, median (range): 3 (2–6)	ORR: 13% (in NSCLC, n = 15)	Completed
**LY6E**	DLYE5953A	I [71]	Solid tumors (incl. NSCLC)	Previously treated not candidates for standard therapy	ORR: 20% (in NSCLC, n = 25)	Completed

Abbreviations: ADC = antibody-drug conjugate; CAB-AXL-ADC = conditionally active biologic anti-AXL antibody-drug conjugate; c-Met = mesenchymal-epithelial transition factor; NSCLC = non-small cell lung cancer; TKI = tyrosine kinase inhibitor; PTK7 = protein tyrosine kinase 7; TfR1/CD71 = transferrin receptor-1; FOLR1 = folate receptor α; CEACAM5 = carcinoembryonic antigen-related cell adhesion molecule 5; NaPi2b = sodium dependent phosphate transporter type II; LY6E = lymphocyte antigen 6, locus E; ORR = Overall Response Rate; PFS = Progression-Free Survival; OS = Overall Survival; mo = months; ILD = Interstitial Lung Disease; AE = Adverse Event; EGFR = epidermal growth factor receptor; EGFRm = EGFR mutated; wt = wildtype; TPS = Tumor Proportion Score; ISAC = Immune-Stimulating Antibody Conjugate; PROTAC = PROteolysis TArgeting Chimera; NS = Not Significant; PR = Partial Response; SD = Stable Disease; CAB = Conditionally Active Biologic; PBC = Platinum-based Chemotherapy; AGA = Actionable Genomic Alteration; ICI = Immune checkpoint inhibitors; mo = month; FDA = Food and Drug Administration.

## 7 The Innovation Pillars of Next-Generation ADCs: New Targets

Recent development has focused not only on tumor antigen expression but also on targeting key pathways linked to tumor aggressiveness, resistance and TME. AXL, ALCAM (CD166), integrin β6, and c-MET represent a class of targets associated with epithelial-mesenchymal transition (EMT), invasion and treatment resistance, including escape from PD-1 blockage. Transferrin Receptor 1 (TfR1) and folate receptor-α (FRA) regulate tumor metabolic pathways such as folate uptake and PI3K/Akt/HIF-1 signaling, while B7-H3 and tissue factor (TF) are implicated in immune evasion and angiogenesis. Eventually, Protein Tyrosine Kinase 7 (PTK7) regulates Wnt signaling and cell differentiation, influencing tumor invasiveness and metastasis [8].

### 7.1 c-MET

Among these, c-MET ADCs have advanced most rapidly. c-MET is dysregulated in NSCLC via overexpression (approximately 25–39%), exon 14 skipping (2–4%) or amplification (5%), the latter linked to resistance in EGFR-mutant disease. Teliso-V consists of an anti-MET antibody linked to MMAE. In the LUMINOSITY trial, a phase II study including patients with EGFR-wildtype (wt) c-MET-overexpressing NSCLC, Teliso-V demonstrated an ORR of 28.6% up to 34.6% in IHC-high tumors, with PFS of 5.7 months. Neuropathy and peripheral edema were the main adverse events [56]. Its recent accelerated approval by the FDA in May 2025 underscores the feasibility of biomarker-driven ADCs in lung cancer; meanwhile, a phase III trial comparing Teliso-V to docetaxel (NCT04928846) is ongoing [72]. In addition, new ADCs targeting MET are currently being tested in phase I clinical studies [73,74,75]. 

ADCs targeting other emerging antigens have also been investigated, including PTK7, B7-H3, integrin β6, TfR1, AXL, FOLR1, CEACAM5, NaPI2b, TF, LY6E, ROR2, ALCAM, 5T4, and LYPD3 (Table 3) [52,53,59,60,61,62,63,64,65,66,67,68,69,70,71,76,77,78,79,80,81]. Some clinical programs have been discontinued due to limited efficacy or safety concerns, as in the case of enapotamab- or lifastuzumab-vedotin [64,68]. Others, instead, showed significant clinical potential. 

### 7.2 PTK7

PTK7 is a pseudokinase enriched in NSCLC with poor prognosis. Cofetuzumab-pelidotin (Cofe-P) consists of an anti-PTK7 antibody conjugated to a microtubule inhibitor. In a phase Ib trial, Cho et al. included 65 patients with NSCLC expressing PTK7 and reported an ORR of 18% and a median PFS of 5.5 months [59]. 

### 7.3 B7-H3

B7-H3 is a transmembrane glycoprotein that promotes immune evasion and tumor progression [8]. MCG018, an ADC targeting B7-H3, was tested in advanced solid tumours and demonstrated moderate safety and efficacy, particularly in metastatic castration-resistant prostate cancer [76,82]. Recently, Ifinatamab-deruxtecan (DS-7300) showed interesting clinical activity in advanced solid tumors [77]. A phase I/II study reported an ORR of 4 (31%) of 13 patients with squamous NSCLC [60]. A phase II study in 183 patients with extensive-stage small cell lung cancer (SCLC) reported an impressive response rate of 48.2% and a median PFS of 4.9 months [61]. 

### 7.4 Integrin β6

Another interesting target is represented by integrin β6, a cell surface receptor implicated in tumor invasiveness. The combination of sigvotatug-vedotin (SV) and pembrolizumab showed an ORR of 57% (4 of 7 patients) in a recent phase I study [62]. Based on these preliminary results, a phase III study is currently comparing this agent to pembrolizumab in patients with PD-L1 high NSCLC (NCT06758401) [62]. 

### 7.5 AXL

AXL is a cell-surface receptor tyrosine kinase that is expressed in several solid tumor types. Increased AXL expression is associated with tumor resistance to chemotherapy, PD-1 inhibitors, molecular targeted therapy, and radiation therapy. Mecbotamab-vedotin (BA3011) is an anti-AXL ADC. BA3011 alone or in combination with nivolumab demonstrated an ORR of 28% [65]. In the case of enapotamab vedotin, which initially showed meaningful activity in 5 of 8 AXL-selected sarcoma PDX models, the effect did not correlate with AXL expression and subsequent findings suggested that the response was likely influenced by additional downstream determinants such as tumor sensitivity to MMAE payloads. These findings point to biomarker and ADC design limitations creating an engineering mismatch between target selection and payload effectiveness [83]. In a phase I/II study, clinical activity likewise did not clearly correlate with AXL expression, further supporting efficacy limiting factors like internalization and/or intracellular release of the cytotoxic payload, as well as a payload resistance mechanism with suggestions that increasing the payload to antibody ratio could potentially mitigate these mechanisms and increase efficacy, particularly among patients with NSCLC, as free MMAE can generate bystander toxicity [84]. The key engineering problem was therefore not merely patient selection, but also the payload design, which is susceptible to heterogeneous delivery and resistance.

### 7.6 CEACAM5

Tusamitamab-ravtansine (TUSA) is an ADC that targets cells expressing CEACAM5. In a phase Ib study including 64 patients with high and 28 patients with moderate CEACAM5 expression and n-sq NSCLC, the confirmed partial responses (PR) were 20% and 7% in high and moderate expressors, respectively. Interestingly, the most common grade 3 treatment-related adverse event was keratopathy [81]. However, Besse et al. found no difference in median OS with tusamitamab-ravtansine vs. docetaxel (12.8 vs. 11.5 months) [53]. An exploratory analysis of patients with long-term treatment exposure has revealed that responses to TUSA could not be related to CEACAM5 expression by IHC [85]. 

### 7.7 Tissue Factor

Eventually, TF, also known as thromboplastin or factor III, is a transmembrane glycoprotein that is overexpressed in many types of cancer. Tisotumab-vedotin is an ADC that targets TF and was approved by the FDA for the treatment of recurrent or metastatic cervical cancer based on the innovaTV 301 trial [86]. The phase I/II trial in patients with advanced or metastatic solid tumors reported an ORR of 13% in 2 of 15 patients with NSCLC [70]. 

## 8 Metabolic and Immunoregulatory Targets and Payloads

The landscape of ADCs is evolving beyond traditional targets on expressing cells. An interesting development of ADCs is to target immune-modulation pathways using immune-stimulatory payloads, creating the so-called immune-stimulating antibody conjugate (ISAC) (Table 4) [87]. Therefore, ADCs can trigger both cytotoxicity and immune activation [39].

Activation of Stimulator of Interferon Genes (STING) in tumor and myeloid cells can trigger innate immunity and type I interferon (IFN) production, thus enhancing immunogenicity in “cold” tumors and promoting immune memory and bystander killing [88,89]. Another example of immune targets is toll-like receptors (TLR). Activation of TLR can stimulate plasmacytoid, dendritic cells and other immune cells, inducing the type I interferon pathway and the production of pro-inflammatory cytokines, eventually leading to an activation of the immune response in immunologically silent tumors and synergizing with ADC-induced cell death [90,91]. 

Preclinical data of an HER2-directed STING agonist ADC, XMT-2056, demonstrated tumor regression in a variety of tumor models with high and low HER2 expression. Importantly, XMT-2056 outperformed the free STING agonist, achieving greater antitumor activity while causing less systemic inflammation. Its mechanism allowed immune-mediated killing of HER2-negative tumor cells, highlighting its potential to overcome heterogeneous HER2 expression. Another peculiar feature of XMT-2056 is that its antibody component does not interfere with trastuzumab or pertuzumab binding. As a result, a similar benefit was observed when combined with T-DXd. Moreover, pairing XMT-2056 with anti-PD-1 therapy promoted the development of immunologic memory, supporting its rationale in combination strategies [92]. Similarly, BDC-1001, an ISAC consisting of a trastuzumab biosimilar conjugated to a TLR7/8 agonist via a non-cleavable linker, demonstrated durable disease control and acceptable safety in heavily pretreated HER2-expressing solid tumors as monotherapy and in combination with nivolumab [93]. 

**Figure 3:**
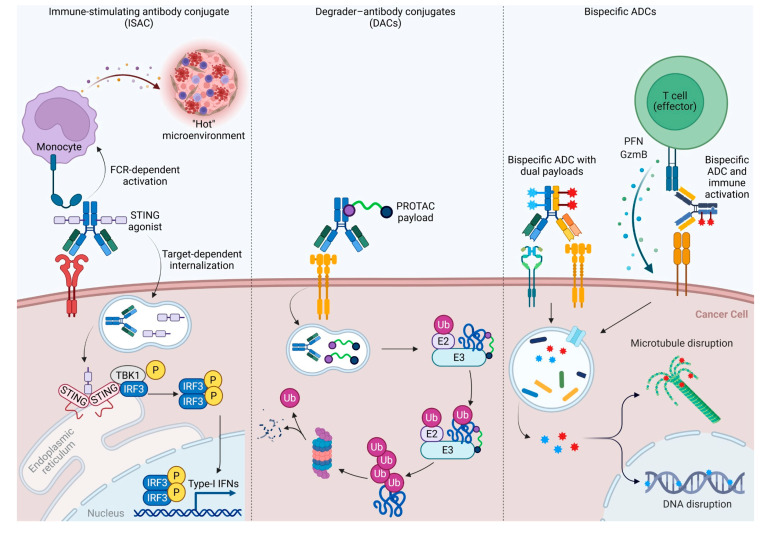
Overview of next-generation ADCs. Immune-stimulating antibody conjugates (ISACs) deliver immunomodulatory payloads (e.g., STING agonist) where target-dependent uptake drives type I interferon (IFN) production within tumor cells, while Fc receptor (FCR)–dependent uptake activates monocytes and myeloid cells, promoting an immune-rich (“hot”) microenvironment. Degrader–antibody conjugates (DACs) use PROTAC payloads to induce selective ubiquitination and proteasomal degradation of oncogenic intracellular proteins. Bispecific ADCs may incorporate dual payloads to deliver complementary cytotoxic mechanisms or engage T-cells, enabling tumor killing through the release of perforin (PFN) and granzyme B (GzmB). This figure was created with Biorender. Abbreviations: STING = stimulator of interferon genes; TBK1 = TANK-binding kinase1; IRF3 = interferon regulatory factor 3; PROTAC = PROteolysis TArgeting Chimera.

An intriguing extension of ADC technology is the possibility of conjugation with PROTACs, Table 4 [87]. PROTACs can induce selective degradation of oncogenic intracellular proteins via E3 ligase, representing an innovative method to overcome resistance from non-mutational target suppression and address potentially undruggable oncogenic proteins [87]. Preliminary results from a phase I/II study of CFT1946 in refractory BRAF-V600 mutant solid tumors found a response rate of 4%, with 7 (50%) of 14 evaluable patients with stable disease [94]. A summary of emerging ADC technologies is illustrated in Fig. 3. 

**Table 4:** Novel immunoregulatory and metabolic targets.

Target	Drug Name	Phase	Patient Population	Key Efficacy Results	Key Safety/Status Notes
**STING Agonist (ISAC)**	XMT-2056	I [92]	HER2-targeted Immune-Stimulating ADC	-	Recruiting
**PROTAC (BRAF V600)**	CFT1946	I/II [94]	BRAF V600 mutant solid tumors (incl. NSCLC)	50% stable disease	Active, not recruiting
**PROTAC (SMARCA2)**	PRT3789	I	SMARCA4-deficient solid tumors	-	NCT05639751; Completed; Selective SMARCA2 degrader
**PROTAC (KRAS G12D)**	ASP3082	I	KRAS G12D-positive solid tumors (including NSCLC)	ORR: 33% (at 300 mg)	NCT05382559; Recruiting

## 9 Combination Strategies and Bispecific ADCs

Resistance to ADCs usually arises from either heterogeneous antigen expression or loss, leading to target down-regulation and incomplete tumor engagement [95]. Donaghy et al. also described the importance of internalization defects and efflux transporter upregulation as barriers to efficacy [33]. To address this, bispecific ADCs have been developed to hit distinct tumor-associated antigens and pathways, maintaining efficacy despite potential adaptive resistance mechanisms [31]. Another area of interest is the combination of ADCs with TKIs, ICIs and other ADCs. Again, such combinations have the potential to overcome resistance but require accurate balancing of overlapping toxicities and interactions [1,31]. 

Combination studies are expanding rapidly in NSCLC (Table 5). In a first attempt, Teliso-V was combined with erlotinib in a phase Ib study demonstrating a 32.1% response rate in EGFR-mutant NSCLC after progression on EGFR-TKI therapy [57]. Subsequently, the combination with osimertinib yielded response rates of more than 50% in patients with advanced c-MET overexpressing NSCLC after failure on prior osimertinib [58]. This increased efficacy is biologically plausible, as MET amplification and overexpression is a known mechanism of acquired resistance to osimertinib, enabling bypass signaling. Targeting c-MET with an ADC therefore provides a strategy to overcome resistance to EGFR TKI [9]. Combination strategies integrating ADCs with ICIs are supported by a strong rationale: ADC can induce the release of damage-associated molecular patterns (DAMPs) from killed cells, leading to dendritic cell activation and antigen presentation, and subsequent engagement of T-cells, eventually enhancing responsiveness to ICIs [96]. Indeed, the phase II EVOKE-2 trial reported encouraging response rates with SG plus pembrolizumab, supporting the ongoing phase III EVOKE-3 study in PD-L1–high NSCLC (NCT05609968). Similarly, the OptiTROP-Lung01 trial demonstrated promising activity for SKB264, a novel TROP2-directed ADC, combined with a PD-1 inhibitor, in PD-L1–high and squamous tumors, with confirmatory phase III trial (NCT06448312) [9]. 

Inspired by urothelial cancer, dual-ADC approaches demonstrated the potential for multi-mechanistic strategies, though toxicity remains a challenge [97]. However, dual-ADC strategies are still early phase in lung cancer. 

As mentioned previously, bispecific and/or dual-payload ADCs are emerging to tackle resistance and enable multi-mechanism strategies, particularly in defined subpopulations such as EGFR-mutant NSCLC. In these patients, acquired resistance frequently involves bypass pathway activation, including MET amplification and HER3 signaling, providing a potential target for cytotoxic payloads [98]. Izalontamab-brengitecan (BL-B01D1) is a bispecific ADC that targets EGFR and HER3 with a topo I inhibitor payload. Early results in heavily pretreated patients with NSCLC were promising, with an ORR of 63% in EGFR-mutant and 44% in EGFR-wt NSCLC [99]. The same bispecific ADC conjugated with an MMAE payload (SI-B001) was tested in a phase I/II study in patients with EGFR/ALK wt NSCLC after progression on ICI plus chemotherapy, showing promising ORR [100]. In addition, the combination with docetaxel achieved an ORR of 31.3%, up to 50% in patients without AGAs, with a disease control rate up to 77% [101]. Recently, the combination of BL-B01D1 with daily osimertinib achieved the impressive ORR of 95%, with two partial responses pending confirmation [102]. Following the path paved by bispecific and immunomodulatory ADCs, DB-1419 represents the first ADC targeting B7-H3 and PD-L1. This drug showed potent killing in lung cancer cell lines with a favorable pharmacokinetic profile [103]. A first-in-human trial is currently ongoing (NCT06554795). 

To add layers of complexity, bispecific ADCs conjugated with two distinct payloads are on their way to clinical use. A recent paper by Wen et al. provided clinical data demonstrating that patients previously treated with a topo I inhibitor-based ADC had only a 15% response rate when switched to another topo I inhibitor ADC, pointing out the existence of a payload class resistance irrespective of target antigen. In this perspective, dual payload ADCs work by combining two cytotoxic agents with distinct mechanisms of action, exploiting the potential to overcome payload resistance. The paper also discussed how appropriate DAR and payload ratios are critical for synergistic purposes, as well as for therapeutic index [95]. It was found that dual-target dual-payload ADCs, combining an MMA and topo I inhibitors, were more effective than single-payload ADCs in inhibiting cell proliferation across multiple cell lines. It also showed superior efficacy in cell models that are normally resistant to ADC [104]. AZD9592 is an ADC with a bispecific antibody targeting EGFR and cMET, conjugated with a topo I inhibitor and an alkylating agent. The compound has demonstrated promising efficacy and safety in adenocarcinoma and squamous NSCLC with or without AGA. A first-in-human study is ongoing [75]. Another example of a bispecific ADC with dual payload is BIO-201. This ADC targets HER2 and TROP2 and demonstrated significant anti-tumor activity. BIO-201 exhibited a remarkable cytotoxic effect both *in vivo* and *in vitro*, in cancers that express either HER2 or TROP2, or both [105]. 

**Table 5:** Bispecific ADCs in NSCLC.

Target	Drug Name	Phase	Patient Population	Key Efficacy Results	Key Safety/Status Notes
**EGFR × HER3**	Izalontamab Brengitecan (BL-B01D1)	I/II [99]	Metastatic EGFRm NSCLC	ORR: 63%	Recruiting; FDA Breakthrough Therapy (Aug 2025)
**EGFR × HER3**	SI-B001 [100]	I/Ib [100]	EGFR/ALK wt NSCLC (SI-B001 + chemo)	ORR: 31% (50% in AGA-negative)	NCT05020457; Active, not recruiting
**EGFR × MET**	AZD9592	I [75]	Metastatic NSCLC (±Osimertinib)	-	Recruiting; Bispecific ADC

## 10 Limitation and Future Perspective

Next-generation ADCs will integrate biomarker-driven precision involving IHC, ctDNA, and molecular profiling, such as single-cell RNA sequencing and spatial proteomics, supported by radiomics. These approaches will also enable dynamic monitoring of resistance, facilitating sequential treatment strategies like ADC-to-ADC, ADC-to-TKI, or combination treatments. AI platforms will play a crucial role in integrating multi-omics data, tumor profiles and libraries to guide target identification, structural refinement (e.g., DAR, linker type) and toxicity prediction [96]. This will further optimize ADC design: nanocarrier-based systems and liposomes are under development to improve tumor accumulation, blood stability and accurate drug release. Functionalization strategies, such as ligand addition, PEGylation and smart release mechanisms, and exploration of novel targets, offer promising opportunities to enhance therapeutic precision and bioavailability [96]. Despite this promising future, the development of ADCs still faces several challenges, including target heterogeneity, antigen loss, poorly understood resistance mechanisms and off-target toxicities. Additional challenges are related to manufacturing complexity, regulatory approval pathways, toxicity management and cost-effectiveness, all of which may limit broader implementation and exacerbate geographic disparities [96]. Furthermore, biomarker exploration remains challenging, as IHC alone is often insufficient to predict response, assays are not uniformly available across institutions, reproducibility remains an issue, and access to advanced technologies required for next-generation biomarkers is still limited. A global coordinated effort is needed to fully exploit the potential of ADCs in clinical practice.

## Data Availability

Data sharing is not applicable to this article as no datasets were generated or analyzed during the current study.

## Abbreviations

HER2/3	Human Epidermal Growth Factor Receptor 2/3
TROP2	Trophoblast Cell Surface Antigen 2
c-MET	Mesenchymal-Epithelial Transition Factor
PD-1	Programmed Cell Death Protein 1
PD-L1	Programmed Death-Ligand 1
EGFR	Epidermal Growth Factor Receptor
B7-H3	B7 Homolog 3 (CD276)
Integrin β6	Integrin Beta-6 (ITGB6)
PTK7	Protein Tyrosine Kinase 7
AXL	AXL Receptor Tyrosine Kinase
FRA	Folate Receptor Alpha (FOLR1)
TfR1	Transferrin Receptor 1 (CD71)
ROR2	Receptor Tyrosine Kinase-Like Orphan Receptor 2
ALCAM	Activated Leukocyte Cell Adhesion Molecule (CD166)
5T4	Oncofetal Antigen 5T4 (TPBG)
CEACAM5	Carcinoembryonic Antigen-Related Cell Adhesion Molecule 5 (CEA)
NaPi2b	Sodium-Dependent Phosphate Transporter 2b (SLC34A2)
Tissue Factor (TF)	Coagulation Factor III (F3)
LY6E	Lymphocyte Antigen 6 Complex, Locus E
LYPD3	Ly6/PLAUR Domain-Containing Protein 3
PI3K	Phosphoinositide 3-Kinase
Akt	Protein Kinase B
HIF-1	Hypoxia-Inducible Factor 1
